# Recent and projected future climatic suitability of North America for the Asian tiger mosquito *Aedes albopictus*

**DOI:** 10.1186/s13071-014-0532-4

**Published:** 2014-12-02

**Authors:** Nicholas H Ogden, Radojević Milka, Cyril Caminade, Philippe Gachon

**Affiliations:** Zoonoses Division, Centre for Food-Borne, Environmental and Zoonotic Infectious Diseases, Public Health Agency of Canada, 3200 Rue Sicotte, Saint-Hyacinthe, J2S 7C6 Québec Canada; Centre Européen de Recherche et de Formation Avancée en Calcul Scientifique, 42 Av Coriolis, F-31057 Toulouse, Cedex 1 France; Institute of Infection and Global Health, School of Environmental Sciences, Roxby Building, University of Liverpool, Liverpool, L69 7ZT UK; Canadian Centre for Climate Modelling and Analysis (CCCma), Environment Canada, 800 rue de la Gauchetière Ouest, Montréal, H5A 1 L9 Québec Canada; Centre pour l’étude et la simulation du climat à l’échelle régionale (ESCER), Université du Québec à Montréal, 201 avenue Président-Kennedy, Montréal, H2X 3Y7 Québec Canada

**Keywords:** *Aedes albopictus*, Geographic distribution, Climate, Climate change, North America, Chikungunya, Dengue

## Abstract

**Background:**

Since the 1980s, populations of the Asian tiger mosquito *Aedes albopictus* have become established in south-eastern, eastern and central United States, extending to approximately 40°N. *Ae. albopictus* is a vector of a wide range of human pathogens including dengue and chikungunya viruses, which are currently emerging in the Caribbean and Central America and posing a threat to North America.

**Methods:**

The risk of *Ae. albopictus* expanding its geographic range in North America under current and future climate was assessed using three climatic indicators of *Ae. albopictus* survival: overwintering conditions (OW), OW combined with annual air temperature (OWAT), and a linear index of precipitation and air temperature suitability expressed through a sigmoidal function (SIG). The capacity of these indicators to predict *Ae. albopictus* occurrence was evaluated using surveillance data from the United States. Projected future climatic suitability for *Ae. albopictus* was obtained using output of nine Regional Climate Model experiments (RCMs).

**Results:**

OW and OWAT showed >90% specificity and sensitivity in predicting observed *Ae. albopictus* occurrence and also predicted moderate to high risk of *Ae. albopictus* invasion in Pacific coastal areas of the Unites States and Canada under current climate. SIG also well predicted observed *Ae. albopictus* occurrence (ROC area under the curve was 0.92) but predicted wider current climatic suitability in the north-central and north-eastern United States and south-eastern Canada. RCM output projected modest (*circa* 500 km) future northward range expansion of *Ae. albopictus* by the 2050s when using OW and OWAT indicators, but greater (600–1000 km) range expansion, particularly in eastern and central Canada, when using the SIG indicator. Variation in future possible distributions of *Ae. albopictus* was greater amongst the climatic indicators used than amongst the RCM experiments.

**Conclusions:**

Current *Ae. albopictus* distributions were well predicted by simple climatic indicators and northward range expansion was predicted for the future with climate change. However, current and future predicted geographic distributions of *Ae. albopictus* varied amongst the climatic indicators used. Further field studies are needed to assess which climatic indicator is the most accurate in predicting regions suitable for *Ae. albopictus* survival in North America.

**Electronic supplementary material:**

The online version of this article (doi:10.1186/s13071-014-0532-4) contains supplementary material, which is available to authorized users.

## Background

The Asian Tiger mosquito *Aedes albopictus* Skuse (1894), is an aggressive diurnal-biting insect that is associated with the transmission of over 20 human pathogens including arboviruses and *Dirofilaria* spp. nematodes [1,2]. *Ae. albopictus* is listed as one of the top 100 invasive species by the Invasive Species Specialist Group and is considered to be the most invasive mosquito species in the world [3,4].

Native to South-eastern Asia, *Ae. albopictus* naturally occurs in a wide range of habitats including coastland, forests, grasslands, urban areas, water courses and wetlands, and has high ecological flexibility being found in densely vegetated rural areas, agricultural areas as well as urban and sub-urban settings. Over the past 30 years this species has been introduced to parts of Europe as well as parts of Africa, Brazil, Central America, the Caribbean, and southern and eastern United States [1]. Its preference for container habitats for breeding, which include used tyres and containers within peri-domestic settings, has promoted its international spread and establishment close to human habitations. International spread is also favoured by its cold-tolerant eggs and capacity to adapt (in terms of diapause of eggs) to temperate environments [2,5].

In nature and/or the laboratory, *Ae. albopictus* is a competent vector for a wide range of viral diseases of significance for human health, including those mostly transmitted human-to-human such as dengue and chikungunya viruses, as well as vector-borne zoonoses such as West Nile virus (WNV), Eastern Equine Encephalitis virus, Rift Valley Fever virus, Cache Valley virus and LaCrosse virus [6]. The capacity of *Ae. albopictus* to feed on a wide range of host species, and to transmit some of these viruses transovarially, enhances its vector potential [6]. The wide range of viruses transmitted by *Ae. albopictus* means that where it invades it can act as an additional vector of endemic viruses, and permit autochthonous transmission or outbreaks of diseases exotic to the location it has invaded. This capacity is illustrated by the outbreak of chikungunya in Italy in 2007, and autochthonous cases of chikungunya and dengue in a number of locations in Europe [7,8]. Chikungunya and dengue have recently emerged/re-emerged in the Caribbean, to and from where there is considerable trade and travel with North America [9,10]. Even where environmental conditions may be suitable for mosquito vectors and transmission, the introduction of infected mosquitoes or infected people is unlikely to result in sustained transmission of these viruses in most of North America for a range of socio-economic reasons including availability of home air conditioning, urban and building design and human behaviour [11]. However, limited outbreaks or autochthonous cases of these diseases are a possibility where *Ae. albopictus* populations have become established. Autochthonously-transmitted exotic vector-borne diseases can have a significant public health impact [12] and there is considerable current need to assess if and where *Ae. albopictus* populations, and by inference limited outbreaks or autochthonous cases of dengue or chikungunya, could occur.

**Figure 1:**
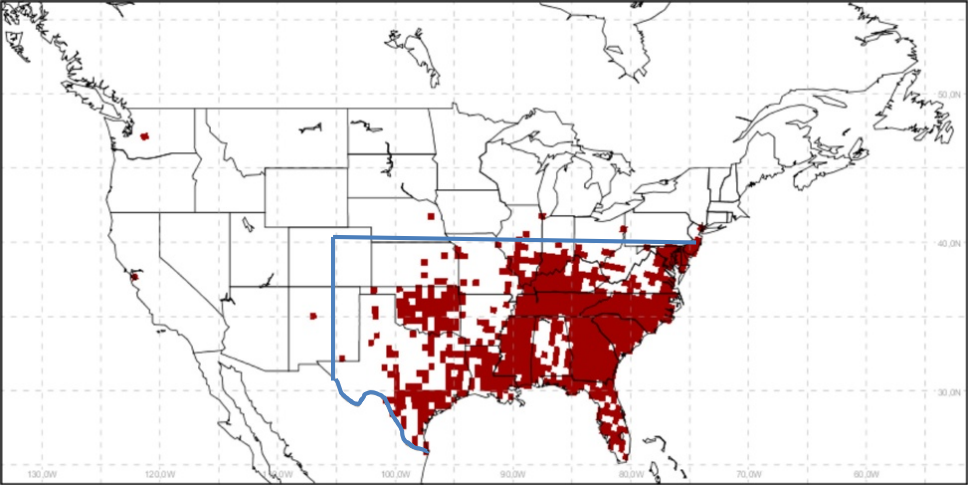
**Surveillance data used in validation of the indicators.** Distribution of *Ae. albopictus* populations in the United States, by county, according to surveillance data compiled by CDC, following transformation into the common 0.44 degrees square grid used in this study. The blue line indicates the data from south of 40°N and east of 105°W used in one of the Receiver Operator Characteristic evaluations of the performance of climatic indicators as described in the Methods section.

A number of studies have aimed to predict where *Ae. albopictus* may be, or invade, under current climate conditions in Europe, Asia and North America, and most identify climate or weather variables (temperature and precipitation) as key determinants of *Ae. albopictus* distribution [6,13-16]. Laboratory-based entomological studies suggest that higher temperatures (accounting for temperature fluctuations [17]), if not associated with increased desiccation, improve conditions for *Ae. albopictus* multiplication, survival and activity [18,19]. It is not unreasonable, therefore, to suggest that a warming climate and changes in precipitation in the context of climate change may drive changes in the geographic distribution of *Ae. albopictus* [20,21]. Only a few studies have to date aimed to predict future possible occurrence of *Ae. albopictus* with climate change [15,22,23], and for North America specifically, to our knowledge there is only one such study, at State-level [24]. *Aedes albopictus* invaded the southern United States, in Texas in 1985, and genetic analyses suggest that the colonising *Ae. albopictus* originated in temperate Japan [6]. *Ae. albopictus* has now thought to have established breeding populations in States that are close to, or border Canada (Figure 1). In Canada, reproducing populations of *Ae. albopictus* are not known to exist at present (although individual mosquitoes of this species have been found during surveillance [25]). In this study we aimed to evaluate the potential of this mosquito species to become established more widely in the United States and Canada under current and future climatic conditions, and provide risk of transmission of chikungunya and dengue that are currently exotic to Canada and most of the United States. We assume that introduction of this mosquito into new geographic regions of the United States and Canada is possible either as a consequence of natural expansion of the mosquito’s range from endemic locations in the United States, or by importation by trade, within-North America or with other parts of the world, in products such as house plants and tyres that have been previously implicated as a means by which this species can be introduced [6,26,27].

## Methods

In this study, indicators of climatic constraints on *Ae. albopictus* population survival, that have been elucidated previously in studies in Asia and Europe, were used to assess the possible current and future distributions of this mosquito in North America.

### Climatological indicators of *Ae. albopictus* survival

In this study we used three climatological indicators of *Ae. albopictus* survival to assess current and future climate suitability over the contiguous United States and Canada. These indicators have been used previously to assess current and future climate suitability in Europe for this mosquito [22].

A criterion used by Caminade *et al.* [22] that included seasonal activity of *Ae. albopictus* in Europe was not considered here because series of day length are not uniformly available over the geographic region studied here. The climatological indicators are described as follows:

i) An indicator (hereafter termed OW) of the suitability of mean air temperature in January (T_jan_) for overwinter survival of *Ae. albopictus* (after Medlock et al. [14]) as well as the occurrence of sufficient annual precipitation (P_ann_) for reproduction. OW provides a four point ordinal scale of suitability for *Ae. albopictus*: (0) very unsuitable if T_Jan_ is lower than 0°C and P_ann_ is below 500 mm, followed by the increasing levels of suitability: (1) moderate when 0°C ≤ T_Jan_ < 1°C and 500 mm ≤ P_ann_ <600 mm, (2) high when 1°C ≤ T_Jan_ < 2°C and 600 mm ≤ P_ann_ <700 mm, and (3) very high when T_Jan_ ≥ 2°C and P_ann_ ≥700 mm.
ii) An indicator (after Kobayashi *et al.* [13]), hereafter termed OWAT, that combines climatic suitability as defined by OW ≥1 with different thresholds of mean annual temperature (T_ann_). OWAT provided five point ordinal scale: (0) very unsuitable conditions when T_ann_ is below 9°C, (1) low risk when 9°C ≤ T_ann_ < 10°C, (2) moderate risk if 10°C ≤ T_ann_ < 11°C, (3) high risk if 11°C ≤ T_ann_ < 12°C, and (4) totally suitable conditions if T_ann_ ≥ 12°C.
iii) An indicator based on the overwintering and summer temperatures expressed through a sigmoidal function [28]. For this indicator January and summer (June–July–August) temperatures were transformed into an interval ranging between 0 and 255 using sigmoidal functions. For precipitation, suitability was zero when annual precipitation was lower than 450 mm and maximum when precipitation was higher than 800 mm. For summer temperatures, suitability was zero when temperatures were lower than 15°C and higher than 30°C and maximum between 20°C and 25°C. For January temperatures, suitability was zero when temperatures were lower than 2°C, and maximum when temperatures were higher than 3°C. These three parameters that were used to define this indicator were then linearly combined (using the arithmetic average) to define a level of suitability of a set of climatic conditions for *Ae. albopictus*. The level of suitability according to this indicator (termed SIG hereafter) was finally rescaled to range between 0 and 100.

### Observed climate data

The observation-based climatic suitability over North America for the period 1981–2010 was derived using station-based gridded daily observations of temperature and precipitation. To cover both the United States and Canada, two independent high-resolution databases were merged. These databases are the CONUS L2013 [29] for the conterminous United States and ANUSPLIN for Canada south of 60°N [30,31], and the merged database is termed ANUSPLIN-CONUSL13 in the following.

### Geographic and temporal representation of current and future climate data

The geographic domain and horizontal mesh resolution varied in size for the gridded observations, and amongst the climate model outputs. Therefore, for consistency in analyses and comparisons, a common grid projection was designated as a regular latitude-longitude grid with a spatial resolution of 0.44 degrees square, extending over North America between 20°N and 60°N.

For observed and projected climate, the climatic indicators of *Ae. albopictus* survival were derived from long-term climatic averages of temperature and precipitation over different time windows. Mean temperatures (January, summer [June-July-August] and annual) were calculated for each year and precipitation was aggregated to annual accumulation and 5-year moving averages for each year were calculated for each climate measure to reduce effects of inter-annual variability.

**Figure 2:**
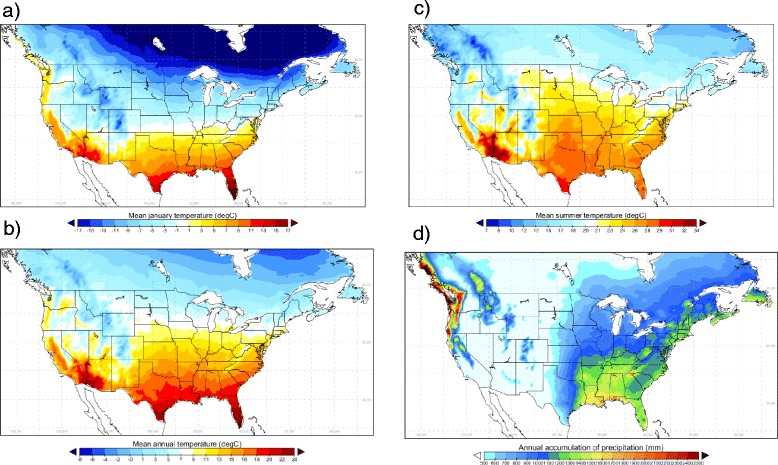
**Current climate data.** Long-term mean values for the period 1981–2010 of **a)** mean January temperature; **b)** mean annual temperature; **c)** mean summer (June, July and August) temperature; and **d)** cumulative annual precipitation for Canada and the United States. Results were obtained from daily time series of temperature and precipitation in the United States and Canada combined into the ANUSPLIN-CONUSL13 dataset and interpolated onto the common 0.44 degrees square grid used in this study.

Values for observed or projected temperature and precipitation for each grid cell were used to classify the grid cells into one of the categories of OW and OWAT, and assign the grid cells a value for SIG. Where climate data and climate model output had a different grid projection to the common grid projection (detailed in Table 1), Inverse Distance Weighting (IDW) interpolation (in Climate Data Operators Version 1.6, Max-Planck-Institut für Meteorologie, Hamburg, Germany) was applied to the raw time series of temperature and precipitation. The observed climate data (ANUSPLIN-CONUSL13) interpolated to the common grid are shown in Figure 2.

**Table 1:** **Selection of RCMs used in this study**

**Acronym**	**Grid projection & Hor. resolution**	**Forcing by global reanalysis**	**Time windows**	**GCM forcing/future scenarios**	**Time windows**
CRCM4.2.3	Polar-Stereographic with true resolution of 45 km at 60°N 180×172 points	ERA-40 [41]	1981 to 2004	CGCM3.1 member 4	Historical 1971 to 2000
		ERA-Interim [43]			*SRES A2* 2011 to 2070
CRCM5*	Rotated Pole latitude-longitude at resolution of 0.44° 172×160 points	″	″	CanESM2	Historical: 1971 to 2005, *RCP4.5/RCP8.5* 2006 to 2070
				*RCP4.5*	
CanRCM4*	Rotated Pole latitude-longitude at resolution of 0.44° 155×130 points	″	1989 to 2010	CanESM2	″
				*RCP4.5*	
				*RCP8.5*	
HIRHAM5*	Latitude-longitude at resolution of 0.44° 130×155 points	″	″	ECEARTH	″
				*RCP4.5*	
				*RCP8.5*	
RCA4-v1*		″	″	″	″
RegCM3**	Transverse Mercator at resolution of 50 km 170×110 points	NCEP/DOE AMIP-II [44]	1981 to 2000	GFDL	Historical 1970 to 1999
					*SRES A2* 2040 to 2069
ECPC**	Polar-Stereographic with true resolution of 50 km at 60°N 116×147 points	″	″	″	″
MM5I**	Lambert Conformal at resolution of 50 km 123×99 points	″	″	CCSM	″
WRF**	Lambert Conformal at resolution of 50 km 134×109 points	″	″	″	″

*Simulations obtained from the CORDEX project; **Simulations obtained from NARCCAP runs.

### Validation of indicators and selection of cut-off values

Data on occurrence of *Ae. albopictus* in surveillance in the United States from 1985–2010 were provided by the United States Centers for Disease Control and Prevention (CDC) at a spatial resolution of county. There is uncertainty in the surveillance data because mosquito surveillance has not been spatio-temporally systematic. While the majority of counties in the United States have undertaken mosquito surveillance during the 1985–2010 period (particularly those of the west coast, the Rocky Mountain region, the upper Midwest, the northeast and the Atlantic coast [32]) false negative counties likely occur due to absence of mosquito surveillance. The main objective of validation was, therefore, to compare amongst the indicators in their power to predict the observed data and establish if any one of them performed particularly well (suggesting more emphasis should be placed on it) or badly (suggesting it should not be used). The surveillance data were disaggregated into the common grid projection used in all analyses in this study (as described above) and a two- dimensional spatial array of zeros and ones was created: a value of 1 was assigned to each grid cell within a county in which the vector had been detected, and zero if not (Figure 1). Receiver Operator Characteristic (ROC) analysis [33] was performed in StataSE11 for Windows (College Station, TX, USA) for each of the climatological indicators using presence-absence of *Ae. albopictus* for each grid cell during 1981–2010 as the outcome. For the ordinal scales of the OW and OWAT indicators, ROC analysis allowed calculation and comparison of the sensitivity and specificity of different categories as cut-off levels for climatic suitability for *Ae. albopictus* survival. For the continuous SIG indicator, ROC analysis generated an index of the predictive power of SIG (the area under the ROC curve: AUC). ROC analysis also generated specificity and sensitivity values for a range of cut-off values for SIG that could be used to select a cut-off value for classifying suitability of different locations for *Ae. albopictus* under future projected climate [34]. The Youden index (defined as *J* = Sensitivity + Specificity – 1 [34]) was calculated for each of 3092 value points on the ROC curve to provide a single scale of values on which to base selection of cut-off values for climatic suitability using SIG. Two cut-off values for climatic suitability when using the SIG indicator were selected. One value chosen was that giving the maximum value for *J*, which gave equal weight to sensitivity and specificity of classification given that surveillance data for the United States was not collected systematically. Therefore false negatives could occur, and identification of *Ae. albopictus* in surveillance could in some circumstances indicate transient individuals rather than permanent reproducing populations of the mosquito. However a second value for *J* was chosen that gave the highest SIG value for specificity when sensitivity was >90% to provide a more risk-averse assessment of future possible *Ae. albopictus* distributions.

The disaggregation of county-level surveillance data into grid cells artificially increased the sample size of the validation data available for assessing performance from 3112 counties in the conterminous United States to 5789 grid cells. To assess the extent to which this affected estimates of the performance of the different indicators in predicting occurrence of *Ae. albopictus* via the area under the ROC curve (AUC), a data set of 3112 grid cells (i.e. a number equal to the number of counties) was selected at random from the total 5789 grid cells. AUC values for OW, OWAT and SIG were then obtained using this reduced 3112 point data set.

Apart from the non-systematic nature of the mosquito surveillance, an additional possible reason for false negative counties in surveillance data is that the mosquito has not yet been introduced even though climatic and other environmental determinants are suitable. To assess the extent to which this possibility could affect estimates of the performance of the different indicators in predicting occurrence of *Ae. albopictus* via the AUC values, the ROC analyses were repeated for a subset of the data south of 40°N and east of 105°W, which comprises the main region of the United States where *Ae. albopictus* has been found (Figure 1).

### *Future projected distributions of* Ae. albopictus

To assess effects of climate change on possible future distributions of *Ae. albopictus,* and evaluate the degree of uncertainty in these projections at a regional scale, projected precipitation and temperature data were obtained from a range of Regional Climate Models (RCMs). The simulations are based on the lateral boundary conditions arising from reanalysis (i.e. a climate or weather model simulation of the past that includes data assimilation of historical observations, see http://reanalyses.org/) for the historical periods or from global climate model (GCM) output for both current and future climate conditions. A time window of 30 years was used to construct climate change scenarios, which, according to availability of RCM output (Table 1), allowed mapping of the climatological indicators of *Ae. albopictus* survival for the 2020s (2011–2040) and/or 2050s (2041–2070). This approach is consistent with current best practice for studies on impacts of projected climate change [35]. The climate simulations from the nine RCMs used in this study (Table 1) were performed within two main project frameworks. Simulations of four RCMs including CanRCM4 and CRCM5 developed respectively by the Canadian Centre for Climate modelling and analysis division of Environment Canada (CCCma/EC) and by the Centre pour l’Étude et la Simulation du Climat à l’Échelle Régionale (ESCER) at the Université du Québec à Montréal, HIRHAM5 of the Danish Meteorological Institute, and RCA4 of the Swedish Meteorological and Hydrological Institute represent the first available output of an ensemble of RCMs within the Coordinated Regional Climate Downscaling Experiment (CORDEX) project covering the North American Domain [36]. Output from four other RCMs (RegCM3, ECPC, MM5I and WRF) that participated in the North American Regional Climate Change Assessment Program (NARCCAP: [37,38]) and simulations from the CRCM version 4.2.3 [39,40] (CRCM4.2.3 runs provided by Ouranos through the CCCma/EC web site) were also considered in our study. In simulations of current climate, RCMs were forced by lateral boundary conditions of the global reanalysis datasets of the European Centre for Medium-Range Weather Forecasts (ECMWF) ERA-40 [41] or ERA-Interim [42,43] or the National Centers for Environmental Prediction (NCEP) Department of Energy (DOE) reanalysis II [44]. Climate change projections were driven by six GCM projections under two greenhouse gas (GHG) emission scenarios (Table 1). The new emissions scenarios developed for the Inter-Governmental Panel on Climate Change (IPCC) AR5 were used in the CORDEX project. The emissions, concentrations, and land-cover change projections are described in the Representative Concentration Pathways RCP4.5 and RCP8.5 [45]. The other five RCMs use the previous, but widely-applied emission scenario IPCC SRES A2 [46]. The A2 scenario describes a very heterogeneous world with high population but slower economic growth than in other scenarios. The RCM scenarios were created for plausible change based on near future (2011 to 2040 in the case of the RCP emission scenarios), and mid-term future (2041 to 2070) climatic conditions. The scenarios A2, RCP4.5 and RCP8.5 are quite similar in terms of GHG equivalent concentrations during the first part of the 21st century but GHG concentrations are greater in A2 and RCP8.5 than in RCP4.5 after 2050 [20,45,47].

Values for projected temperature and precipitation for each grid cell were used to classify the grid cells into one of the categories of OW and OWAT, and assign the grid cells a value for SIG. Where RCM output had a different grid projection to the common grid projection IDW interpolation was applied to the raw time series of temperature and precipitation.

## Results and discussion

### Validation of indicators and selection of cut-off values

Each of the three indicators for climatic suitability for *Ae. albopictus* performed well in predicting observed *Ae. albopictus* distributions in the United States, whether using the entire (5789 values) dataset of grid cell values or the reduced (3112 values) dataset (Table 2). For OW, when using a cut-off for prediction of absence and presence between points 0 and 1 on the ordinal scale, sensitivity and specificity of prediction of *Ae. albopictus* occurrence were both >90%. For OWAT, performance was best when using a cut-off for prediction of absence and presence between points 2 and 3 on the ordinal scale, where sensitivity and specificity of prediction of *Ae. albopictus* occurrence were 90.2% and 92.2% respectively. For SIG, the area under the ROC curve, when using the reduced dataset, was 0.925 (95% confidence interval 0.913 to 0.936) indicating that SIG was “highly accurate” (using the terminology of Greiner et al. [34]) in discriminating suitable and non-suitable climate for *Ae. albopictus* according to the surveillance data. The different indicators of climatic suitability for *Ae. albopictus* all performed well in comparison with other studies on prediction of *Ae. albopictus* distributions in other parts of the world and/or using different modelling methods [22,23], although AUC values for SIG were greater than those for OWAT, and lowest of OW. However, the uncertainties inherent in the surveillance data mean that inter-study comparisons of the ROC AUC values obtained here should be not be over-interpreted.

**Table 2:** **Data on the performance of the different indicators in discriminating suitable and non-suitable climate for** ***Ae. albopictus*** **according to surveillance data collected in the United States from 1999-2011**

**Indicator**	**Cut-off**	**Sensitivity (%)**			**Specificity (%)**			**% Correctly classified**		
		**A**	**B**	**C**	**A**	**B**	**C**	**A**	**B**	**C**
OW	0 versus ≥1	90.51	90.25	93.18	90.15	90.41	45.83	90.19	90.39	68.83
	≤ 1 versus ≥2	83.28	84.12	85.43	92.35	92.59	58.57	91.31	91.61	71.61
	≤ 2 versus ≥3	72.89	71.59	75.35	93.83	94.12	65.45	91.43	91.52	70.26
	AUC	A = 0.912 (95% CI 0.900-0.9240), B = 0.912 (95% CI 0.895-929), C = 0.742 (95% CI = 0.718-0.766)								
OWAT	0 versus ≥1	90.51	90.25	93.18	91.32	91.57	39.97	91.22	91.42	65.81
	≤ 1 versus ≥2	90.51	90.25	93.18	91.59	91.86	41.29	91.47	91.68	66.49
	≤ 2 versus ≥3	90.21	89.97	92.71	92.18	92.52	43.19	91.95	92.22	67.24
	≤ 3 versus ≥4	89.31	88.86	91.94	92.60	92.95	45.53	92.23	92.48	69.07
	AUC	A = 0.914 (95% CI 0.903-0.926), B = 0.915 (95% CI 0.898-0.931), C = 0.688 (95% CI 0.666-0.709)								
SIG	Cut point 64.64	90.06	89.14	91.47	78.75	80.13	41.29	80.05	81.17	65.66
	Cut point 66.69	84.49	83.29	88.84	92.18	92.88	45.10	91.29	91.77	66.34
	AUC	A = 0.925 (95% CI 0.916-0.934), B = 0.925 (95% CI 0.913-0.936), C = 0.709 (95% CI 0.681-0.737)								

Values indicated by the letter A were obtained using data obtained from the full grid of climate data, Values indicated by the letter B were obtained using a reduced database of grid cells with the same sample size (3112) as the numbers of United States counties in the original surveillance data. Values indicated by the letter C were obtained using only data from south of 40°N and east of 105°W. *AUC* = Area under the Receiver Operator Characteristic curve; *CI* = Confidence interval.

When using the data from south of 40°N and east of 105°W, sensitivity of all of the indicators was high (>90%) when using the cut-off values described above. However, AUC values were reduced to approximately 0.7 for all indicators due to low specificity (<50%, Table 2). This was anticipated because while the selected area is where most *Ae. albopictus*-positive locations have been found, it is also an area where there is likely a particularly high number of counties where mosquito surveillance has not occurred and false negative counties occur [32].While the occurrence of false negative locations may affect AUC values, they should not, however, affect comparisons amongst the indicators. Using the data from south of 40°N and east of 105°W, the order of performance of the indicators changed compared to those obtained using the full data, with OW having the highest AUC values, SIG having the second highest and OWAT the lowest values (Table 2). This suggests that it would be prudent not to reject the possibility of risk of *Ae. albopictus* becoming established in a particular location on the basis of the findings of only one indicator, and to determine risk using output from all indicators.

### *Geographic extent of possible current distributions of* Ae. albopictus

**Figure 3:**
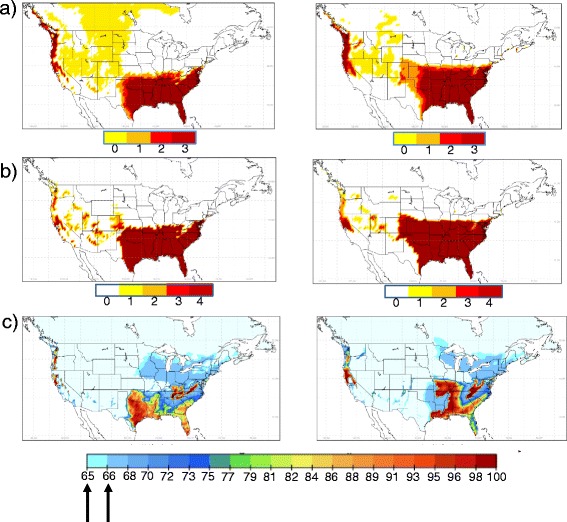
**Predictions of current climate suitability for** ***Ae. albopictus.*** Maps of climatic suitability for *Ae. albopictus* using OW, OWAT and SIG (respectively maps **a**, **b**, and **c**) using observed climate data (1981–2010: left hand column) and CanRCM4 model output for a similar time period (1989–2010; right hand column). The colour scale below each map shows the value for each indicator, and for SIG the cut off at 66.69% and 64.64% is indicated by arrows. For the OW maps, climate of low suitability for *Ae. albopictus* is indicated by both yellow areas (where T_Jan_ is below 0°C and P_ann_ is below 500 mm) and white areas (where both T_Jan_ is below 0°C or P_ann_ is below 500 mm).

Climatic suitability maps for *Ae. albopictus* using OW, OWAT and SIG from observed climate data and one representative RCM (CanRCM4) are shown in Figure 3. The OW and OWAT indicators suggested similar geographic regions of suitable climate including one block of similar, very high climatic suitability extending from Florida to approximately 40° N (with more northerly regions on the Atlantic coast, including Long Island, being suitable) and 105° W in the United States (Figure 3). The western parts of Pacific coast States and Provinces from mid-California to southern coastal British Columbia were also mostly of very high climatic suitability (Figure 3). There were some slight differences with a smaller geographic extent of climatic suitability in States and Provinces bordering the Pacific coast using the OWAT indicator compared to the OW indicator, and the OWAT indicator identified some patches of climatic suitability in States between the mid-west and the Pacific coastal States that were not identified by the OW indicator. The SIG indicator identified a block of climatic suitability extending from Florida to approximately 40° N (with more northerly regions on the Atlantic coast, including Long Island, being suitable) and westwards to 105° W in the United States that was similar to that predicted by OW and OWAT. The SIG indicator also identified western parts of Pacific coast States and Provinces from mid-California to southern coastal British Columbia as climatically suitable although these areas were of geographic limits similar to those predicted by OWAT. In contrast to OW and OWAT, the SIG indicator predicted climatic suitability for regions of more northern States east of approximately 97° W to the Atlantic coast, and northward into southern Ontario, Quebec and the Maritimes in Canada (Figure 3). This was because the values of the SIG indicator are not constrained to zero by sub-zero January temperatures, and because of suitable rainfall and summer temperatures in these regions (Figure 2). With the exception of a few regional differences, the re-analysis outputs of the RCM output for 1989–2010 produced similar patterns of climatological suitability, for each of the three indicators, to values obtained using observed data (Figure 3).

Therefore, in summary, predicted climate suitability using OW and OWAT was similar and conservative, suggesting that the only part of the United States at risk of *Ae. albopictus* populations is that where this mosquito is known to have become established (in the south-eastern corner of the continent) or on the Pacific coast where the mosquito is known to have become established in the past and actively eradicated (such as Washington State [48]). In contrast, the SIG indicator suggested additional climatic suitability in northern states in the eastern United States and in southern Ontario, Quebec and the Maritimes in Canada. If the current northern limit of *Ae. albopictus* populations is well described by the surveillance data in the United States, then SIG likely overestimates risk further north and OW or OWAT are more useful criteria for describing risk of *Ae. albopictus* populations becoming established. However, if *Ae. albopictus* populations do occur north of the limit observed to date in surveillance, then SIG may be the more useful criterion on which to assess risk in northern United States and Canada. Some observations in the field suggest that OW and OWAT do not under-estimate risk. In Italy, the equivalent of OWAT cut-off point 2 best described *Ae. albopictus* population distributions [15] and in experimental studies in Connecticut just north of the northern limit of climatic suitability according to OW and OWAT criteria, *Ae. albopictus* populations failed to become established at least in part due to failure to overwinter [49]. At the northern edge of the geographic range of *Ae. albopictus* according to the surveillance data, at least at one location ongoing field studies support the idea that the presence of *Ae. albopictus* in surveillance data equates with the presence of reproducing populations of the mosquito [50]. Furthermore, niche modelling studies using global *Ae. albopictus* distribution data produce a similar pattern of distribution in North America similar to that predicted using the OW and OWAT indicators [23]. However, because surveillance for *Ae. albopictus* is not, and has not been, systematic in space and time over the United States and in Canada, the true extent of false negative locations in the surveillance data is unknown.

Other factors may limit the predictive power of the methods used here. First, and in common with all “pattern matching” predictive modelling techniques, *Ae. albopictus* populations are spreading in the United States and the observed surveillance data likely represent the current “realised niche” rather than the full theoretical climatological niche width for this species [51]. Evolution of photoperiodic responses of mosquitoes, including of *Ae. albopictus* populations in the United States, has been observed and these (particularly development of egg diapause over winter) may be critical to *Ae. albopictus* population survival in the northern parts of its range in the United States [5,52]. The possibility of such evolutionary adaptations to changing climatic conditions could call into question the validity of assessing future distributions on those observed in the past by changing the climatological niche width. However, northern populations of *Ae. albopictus* have likely already evolved the over-winter survival-enhancing trait of egg diapause [5] and that trait should be accounted for in the surveillance data used here. Interactions amongst mosquito species (particularly competition) likely will also impact the realised niche width and the validity of projections here (e.g. [53]), and are illustrative that the range of ecological determinants of environmental suitability for vectors extends beyond climatic suitability.

### *Geographic extent of projected future distributions of* Ae. albopictus

**Figure 4:**
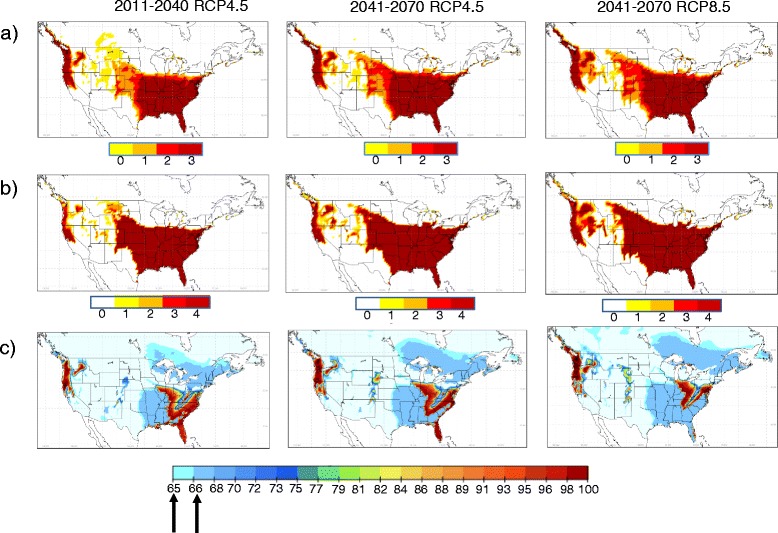
**Projected climatic suitability for** ***Ae. albopictus*** **with climate change.** Future climatic suitability for *Ae. albopictus* using OW, OWAT and SIG (respectively maps **a**, **b**, and **c**), projected using CanRCM4 model output as an illustration. The colour scale below each map shows the value for each indicator, and for SIG the cut off at 66.69% and 64.64% is indicated by arrows. The left hand column shows projections for 2011–2040 and the middle and right hand columns shows projections for 2041–2070 using, respectively, emissions scenarios provided by Representative Concentration Pathways RCP4.5 and RCP8.5. For the OW maps, climate of low suitability for *Ae. albopictus* is indicated by both yellow areas (where T_Jan_ is below 0°C and P_ann_ is below 500 mm) and white areas (where either T_Jan_ is below 0°C or P_ann_ is below 500 mm).

**Figure 5:**
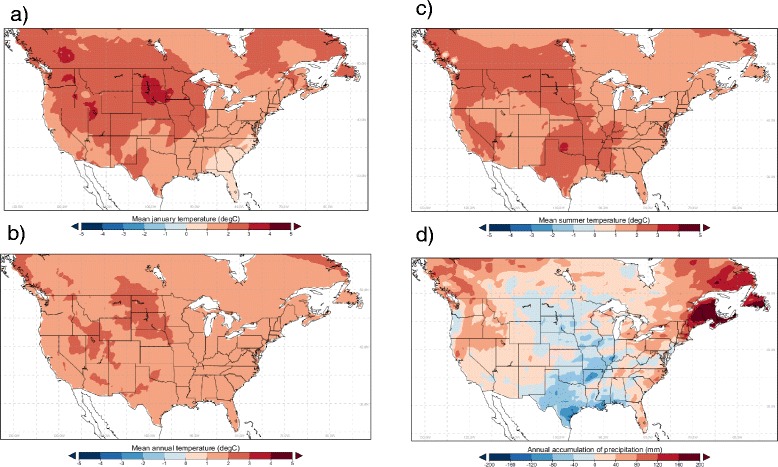
**Projected changes in climate.** An example of projected changes in temperature (panel **a**: average minimum for January; panel **b**: mean annual temperature; panel **c**: mean summer temperature) and precipitation (panel **d**: cumulative annual precipitation) data for Canada and the United States for 2011–2040 relative to 1981–2000 using output from the model CRCM4.2.3.

Projections of potential future climatic suitability for *Ae. albopictus* using output from CanRCM4 are shown as an illustration in Figure 4. Projections of future climatic suitability from all models are presented in Additional file 1. Projected changes in mean annual temperatures and annual precipitation are shown for reference in Figure 5. Projected northward expansion of the geographic range of *Ae. albopictus* (by approximately 200–500 km) was modest using OW and OWAT (Figure 4, Additional file 1), at least in comparison to the range spread projected for other arthropod vectors with climate change [54]. The degree of northward range expansion using OW and OWAT was of a similar magnitude for all RCMs although the current and projected final northern limits by 2041–2070 varied amongst the RCMs. The most northerly projected climatically suitable locations were in Montana, North Dakota, Wisconsin, Michigan, Philadelphia, New York State and New England States in the United States and coastal British Columbia, southern Ontario, Quebec and the Maritimes in Canada (Figure 4, Additional file 1). These northern limits were only projected by climate models that projected the most northern possible distribution of climatic suitability under current climate (e.g. CanRCM4, RCA4, Additional file 1). The models that projected more southerly northern limits under current climate (e.g. CRCM4.2.3, ECPC and MM5I, Additional file 1) projected more southerly northern limits (except along the Pacific coast) with only small projected localised areas of climatic suitability in central and eastern regions of northern United States and southern Canada in the future (Figure 4, Additional file 1). A finding of moderate projected range expansion is consistent with region-scale projections for *Ae. albopictus* in the north-eastern United States using climatological niche predicted using maximum entropy methods [24].

**Figure 6:**
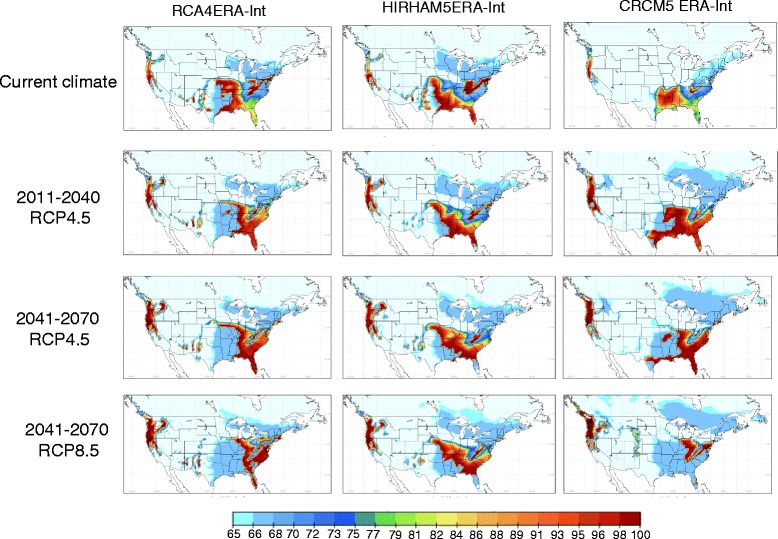
**Variation in climate model output.** An illustration of variation in current (1981–2010) and future (2011–2040 and 2041–2070) projected climatic suitability (using SIG) for *Ae. albopictus* using output from three climate models (from left to right hand columns: RCA4, HIRHAM5 and CRCM5). Projections for the time period 2041–2070 using emissions under both RCP4.5 and RCP8.5 are shown. The colour scale below each map shows the value SIG with the cut off at 66.69% and 64.64% indicated by arrows.

Using the SIG indicator, the projections for future climate suitability in the United States and Canada eastward from 100° W were more complex compared to projections using the OW and OWAT indicators. Using this indicator northward expansion was more extensive (up to 1000 km) into Canada (Figure 4), a band of unsuitable climate dividing the climatically suitable region of the United States was apparent, and climatic suitability in Texas, Louisiana, Oklahoma and Arkansas was reduced due to a combination of reduced rainfall and increased summer temperature (Figure 5). The SIG indicator also predicted more extensive future climate suitability in States along the United States Pacific coast and southern British Columbia compared to OW and OWAT indicators and predicted a future area of climatic suitability in the eastern foothills of the southern Rocky Mountains (Figure 4, Additional file 1). As for the OW and OWAT indicators, there was variation amongst the RCMs in the extent of the future projected northern limit of climate suitability (Figure 6). It could be argued that the SIG overestimates risk by not accounting for absolute limits on population establishment associated with very cold temperatures in winter and very dry conditions. However, perhaps SIG provide a better indication of the climatic suitability in some urban and sub-urban areas where refugia from extremes of climate, including very low mid-winter temperatures, may exist [51,55].

Throughout, variations in projections were greater amongst models than amongst emissions scenarios (Figures 4 and 6, Additional file 1). This would be expected as the RCPs are quite similar to one another during first part of the 21^st^ century as described above, while RCM simulations differ due to differences in their sub-grid scale processes or parameterizations, and differences in their GCM driving conditions (e.g. [56]). Further work is needed to evaluate and quantify the uncertainties arising from variation amongst different RCM outputs, and the power of RCMs to identify suitable temperature and precipitation conditions that are sensitive to more local scale forcing or regional-scale influences not taken into account in the geographic scale of the present study.

## Conclusions

In this study, the OW and OWAT indicators currently seem to offer the best fit to existing data on *Ae. albopictus* distribution in the United States, but predictions of climatic suitability using SIG must be considered as surveillance to date has not been geographically systematic and consistent. If OW and/or OWAT are the most accurate indicators of climatic suitability for *Ae. albopictus* then the possibility of geographic range expansion of this species under current and mid- and long-term future climate is relatively limited with the possible exception of locations along the Pacific coast. However if SIG is the more accurate indicator of climatic suitability for *Ae. albopictus* then more geographically widespread expansion of the northern range of this species may occur where other environmental determinants allow the mosquito to become established. Additional systematic field studies and surveillance will be needed, therefore, to identify which climatic indicator is the most accurate at predicting climate suitable for *Ae. albopictus* and more accurately define the climatic and other environmental determinants of this mosquito [57] to better model and predict its current and future geographic distributions.

## Additional file

Additional file 1:
**The complete set of projections of climate suitability for** ***Aedes albopictus*** **in North America.** Projected climate suitability according to each of the three climatic indicators OW, OWAT and SIG was obtained using output from nine Regional Climate Models (RCMs) as described in the main manuscript text. Maps of RCM-predicted current climate suitability obtained by forcing using global reanalysis, and future climate suitability for the time slices 2011-2040 and 2041-2070 (obtained using Representative Concentration Pathways RCP4.5 and RCP8.5) are shown.

## Abbreviations

AR5: Fifth assessment report of IPCC
AUC: Area under the ROC curve
CCCma/EC: Canadian centre for climate modelling and analysis/environment Canada
CORDEX: Coordinated regional climate downscaling experiment
DOE: Department of energy
ECMWF: European centre for medium-range weather forecasts
ESCER: Centre pour l’Étude et la Simulation du Climat à l’Échelle Régionale
GCM: Global climate model
GHG: Greenhouse gas
IDW: Inverse distance weighting
IPCC: Inter-governmental panel on climate change
NARCCAP: North American Regional climate change assessment program
NCEP: National centers for environmental prediction
RCM: Regional climate model
RCP: Representative concentration pathway
ROC: Receiver operator characteristic
SRES: Special report on emissions scenarios
