# Supplementary material for: Recent and projected future climatic suitability of North America for the Asian tiger mosquito Aedes albopictus
Source: Parasit Vectors. 2014 Dec 2;7:532. doi: 10.1186/s13071-014-0532-4 (PMC4261747; doi:10.1186/s13071-014-0532-4)

Current climate 1981-2010: OW indicator

CRCM5 ERA-Int

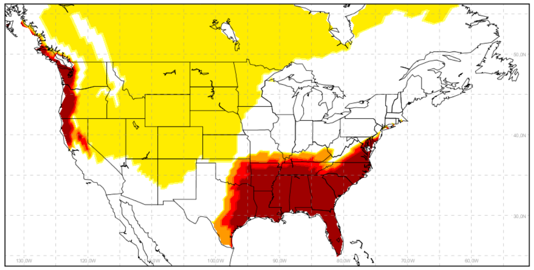

RCA4 ERA-Int

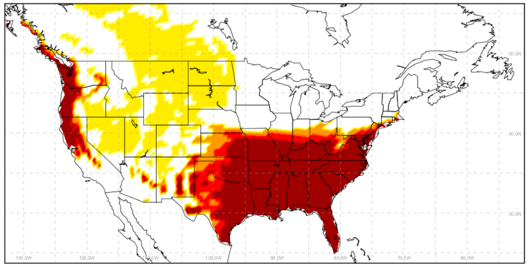

CANRCM4 ERA-Int

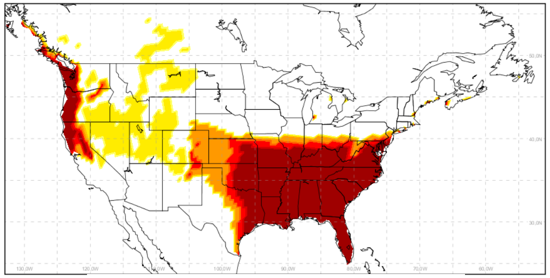

ECPc NCEP/DOE

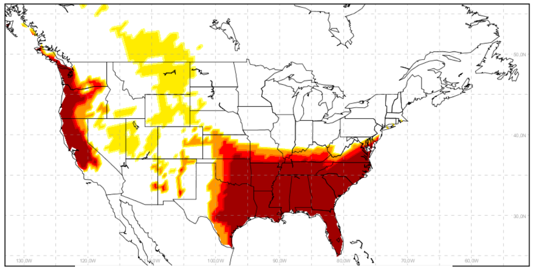

RCM3 NCEP/DOE

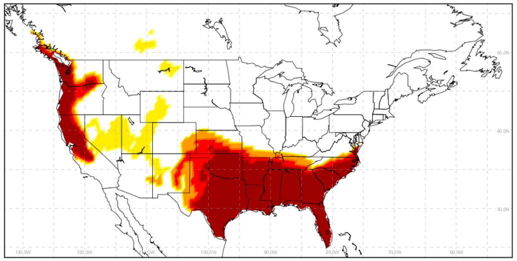

CRCM4.3.2 ERA40

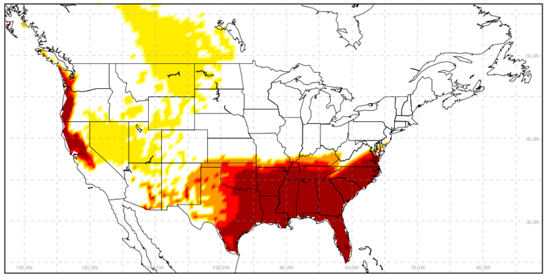

HIRHAM5 ERA-Int

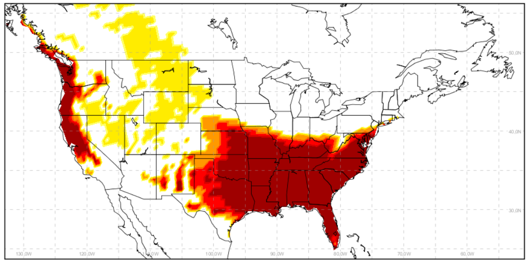

WRGF NCEP/DOE

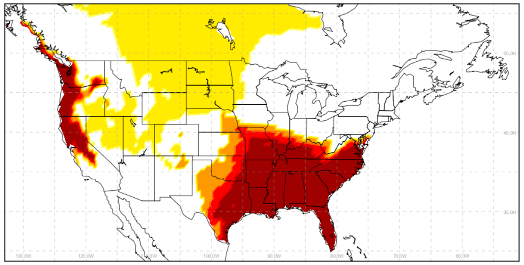

MM5I NCEP/DOE

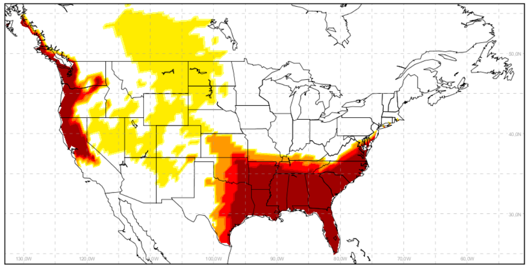

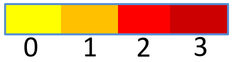

OW value

Projections 2011-2040:
OW indicator

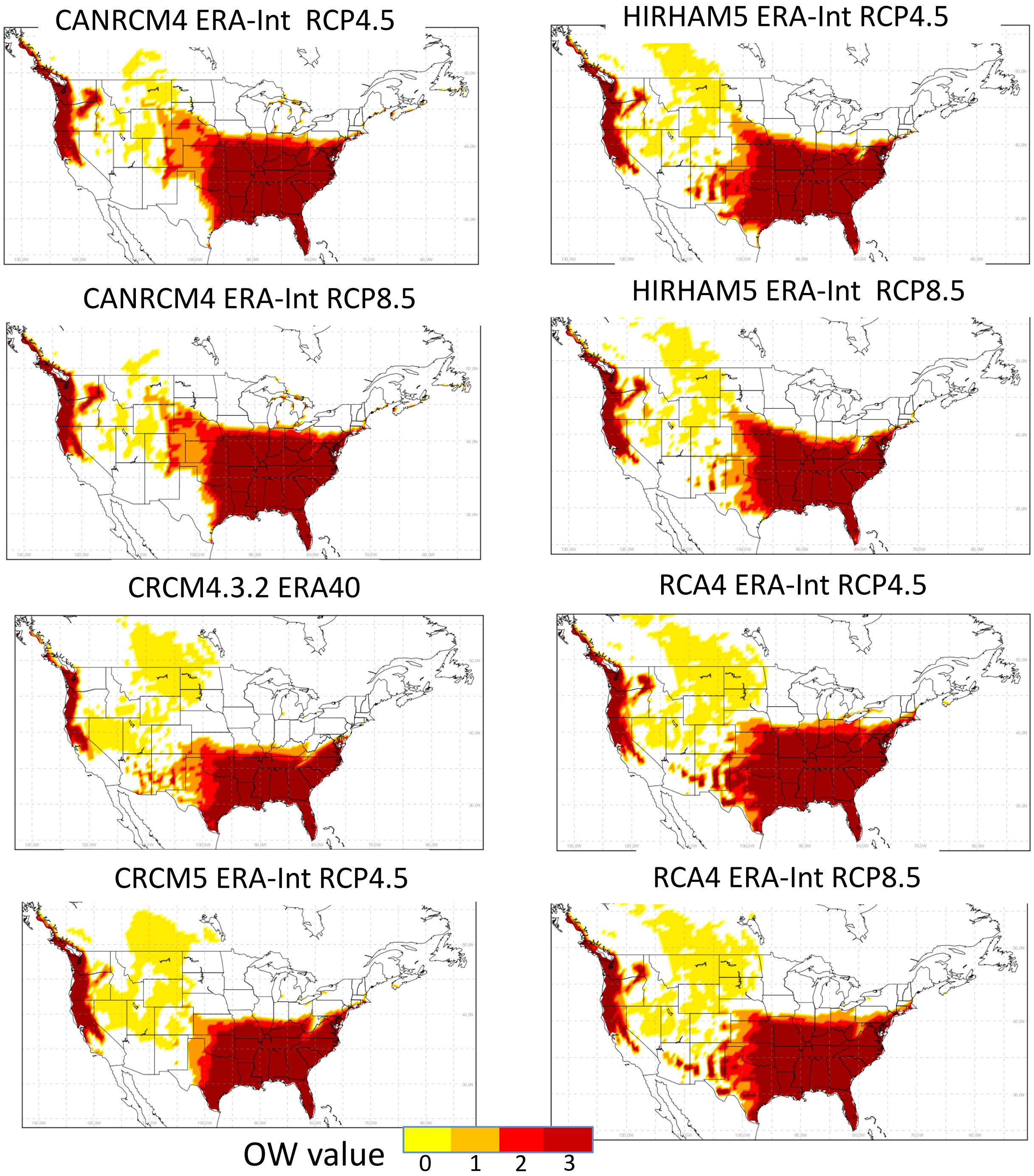

Projections 2041-2071: OW indicator

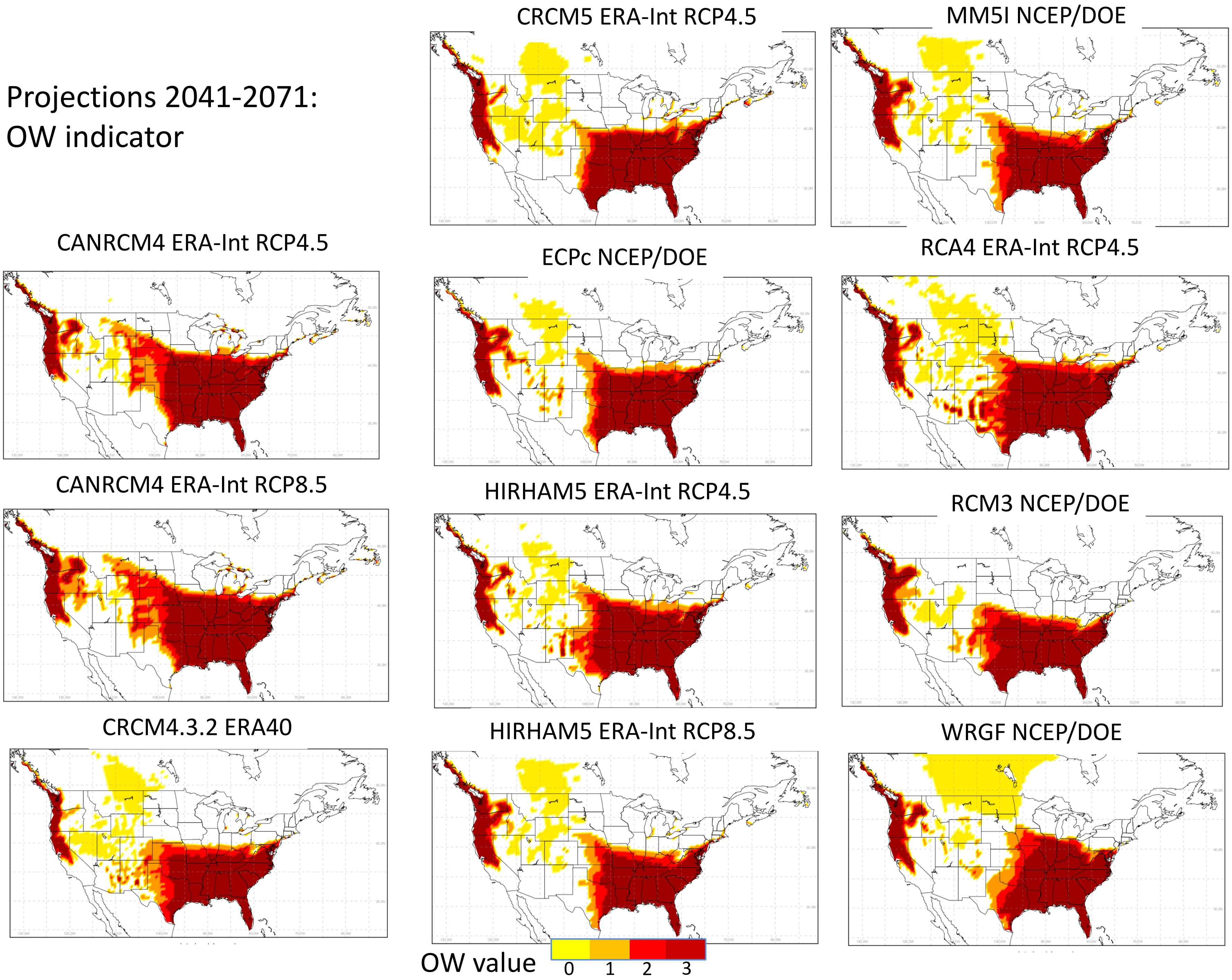

Current climate 1981-2010: OWAT indicator

CANRCM4 ERA-Int

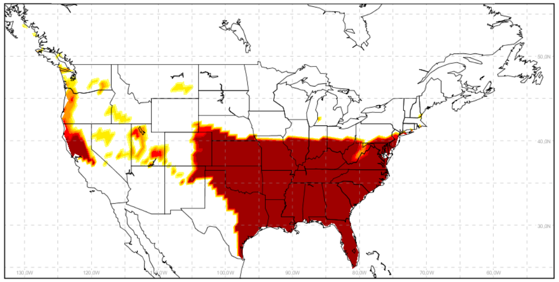

CRCM4.3.2 ERA40

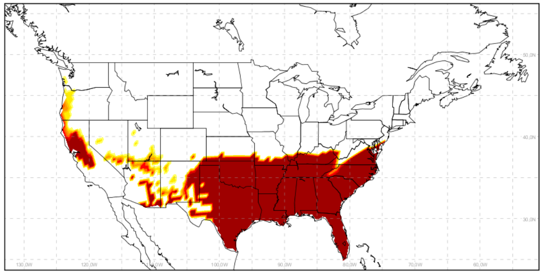

CRCM5 ERA-Int

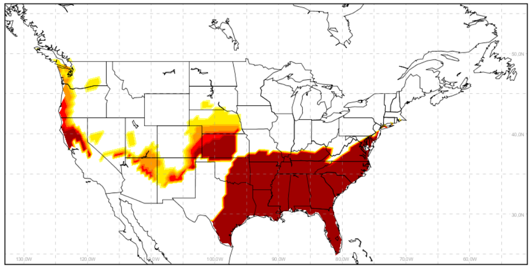

ECPc NCEP/DOE

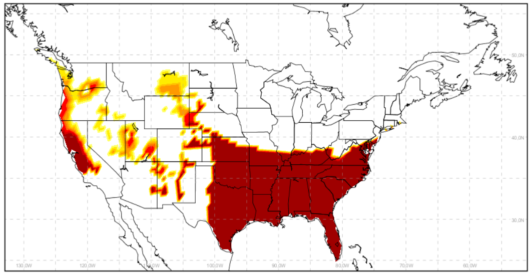

HIRHAM5 ERA-Int

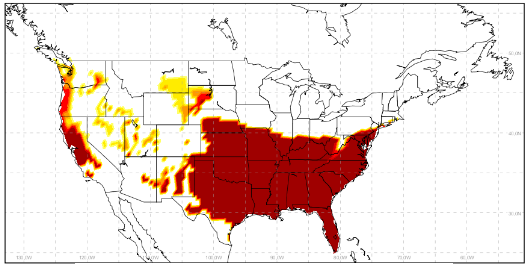

MM5I NCEP/DOE

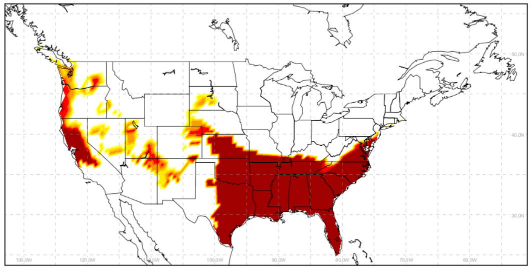

RCA4 ERA-Int

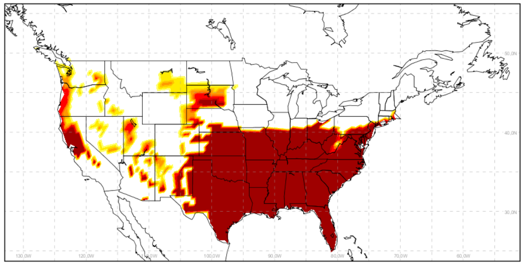

RCM3 NCEP/DOE

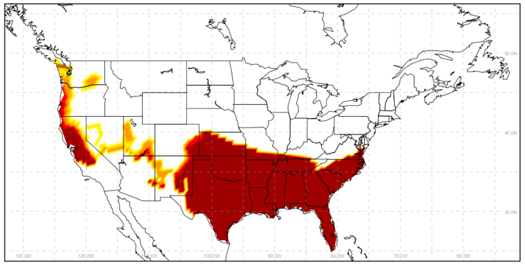

WRGF NCEP/DOE

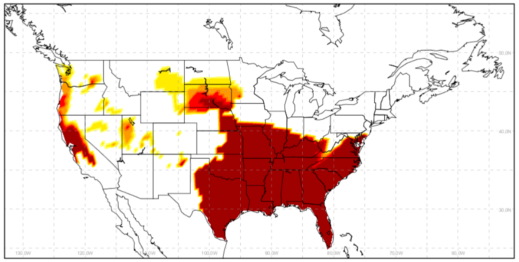

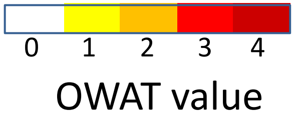

Projections 2011-2040:
OWAT indicator

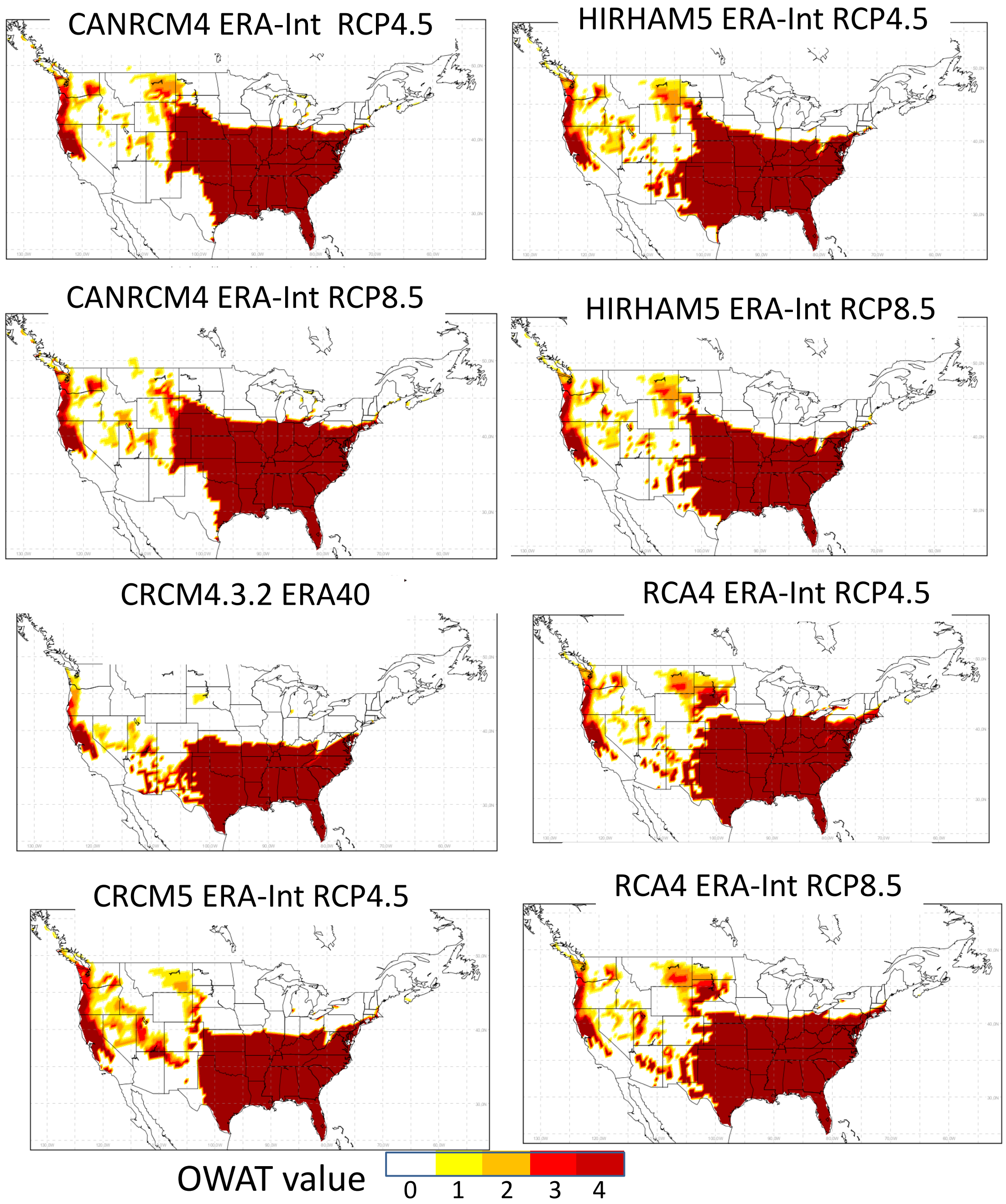

Projections 2041-2071: OWAT indicator

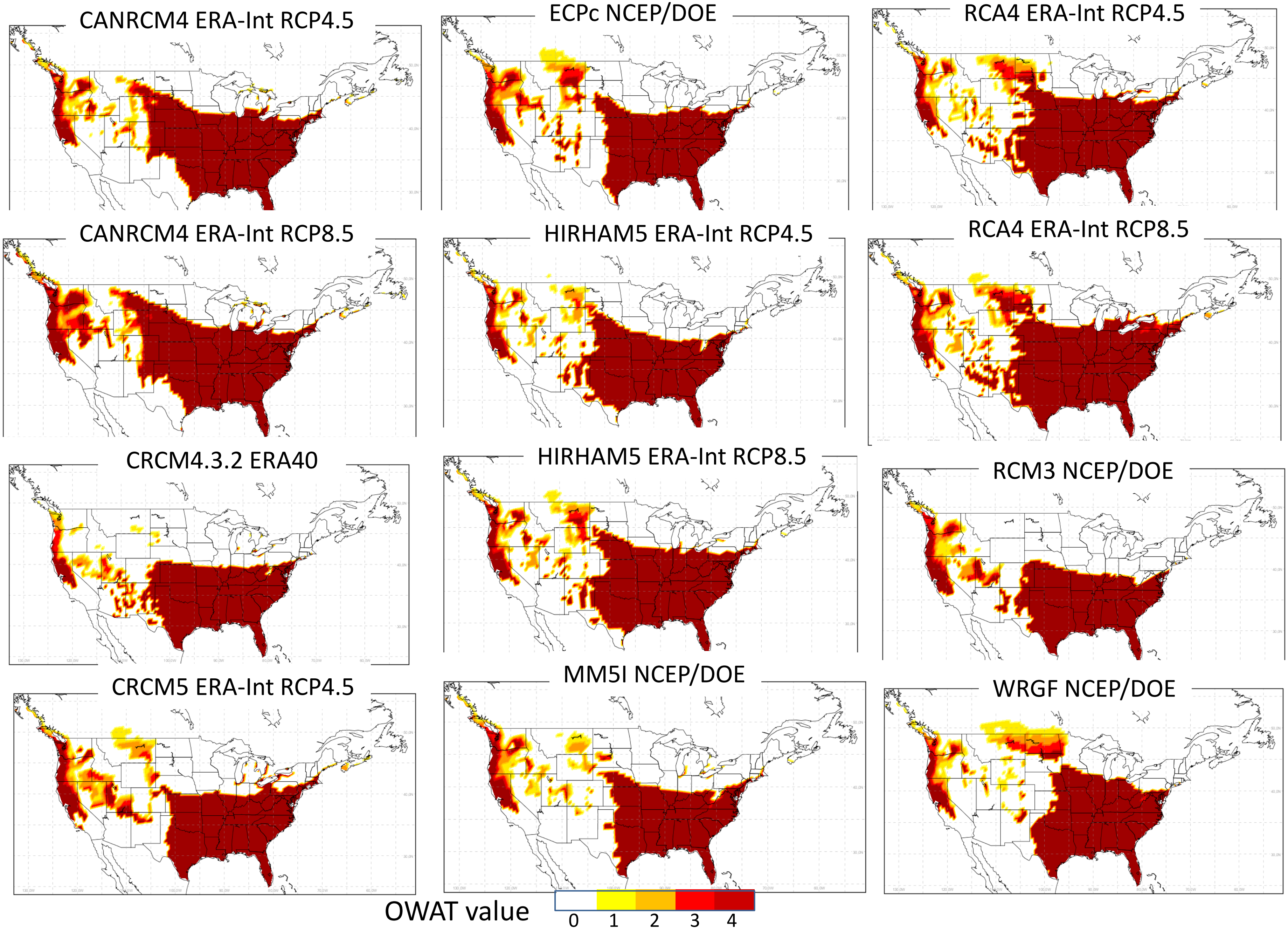

Current climate 1981-2010:
SIG indicator

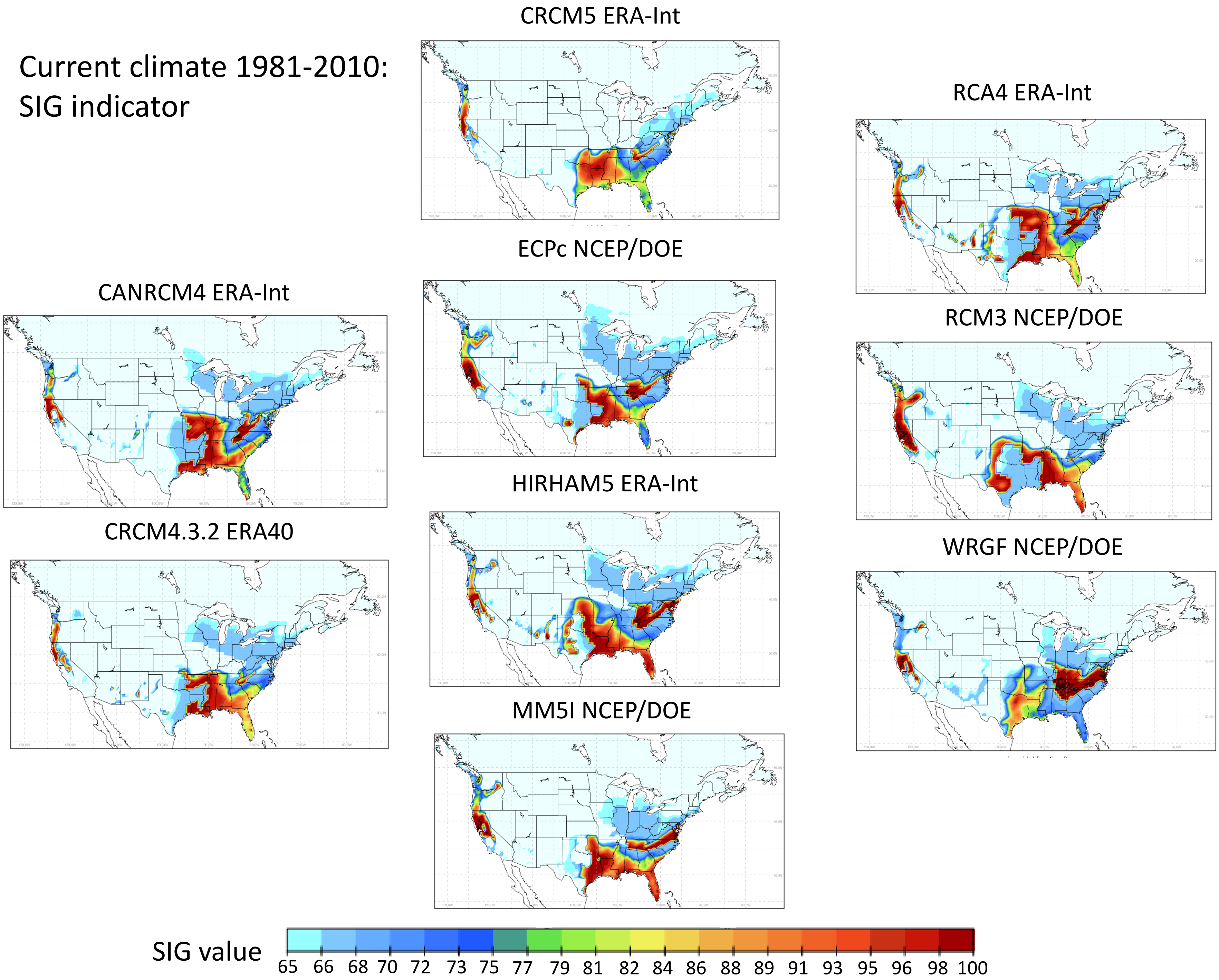

Projections 2011-2040:
SIG indicator

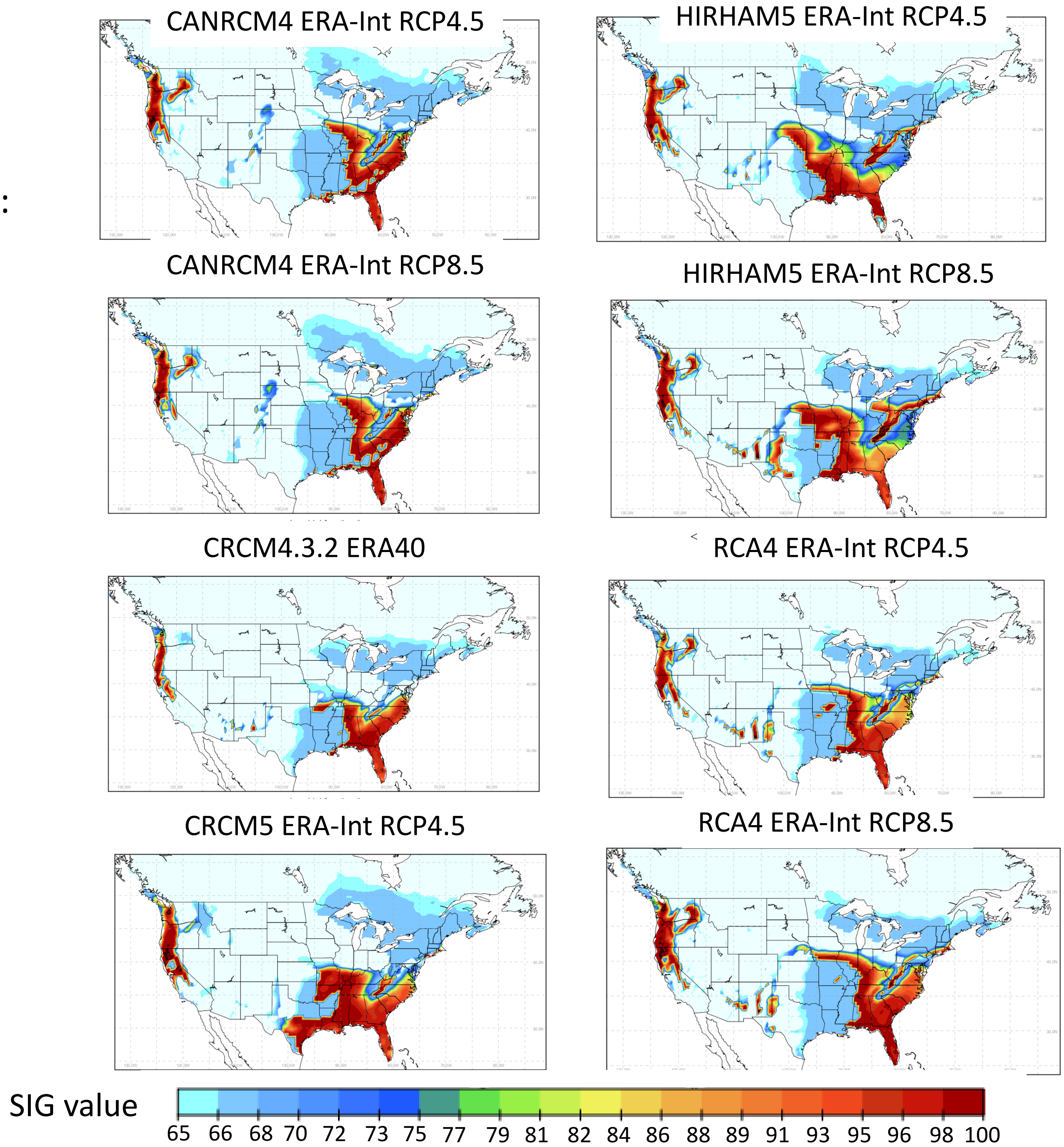

Projections 2041-2070: SIG indicator

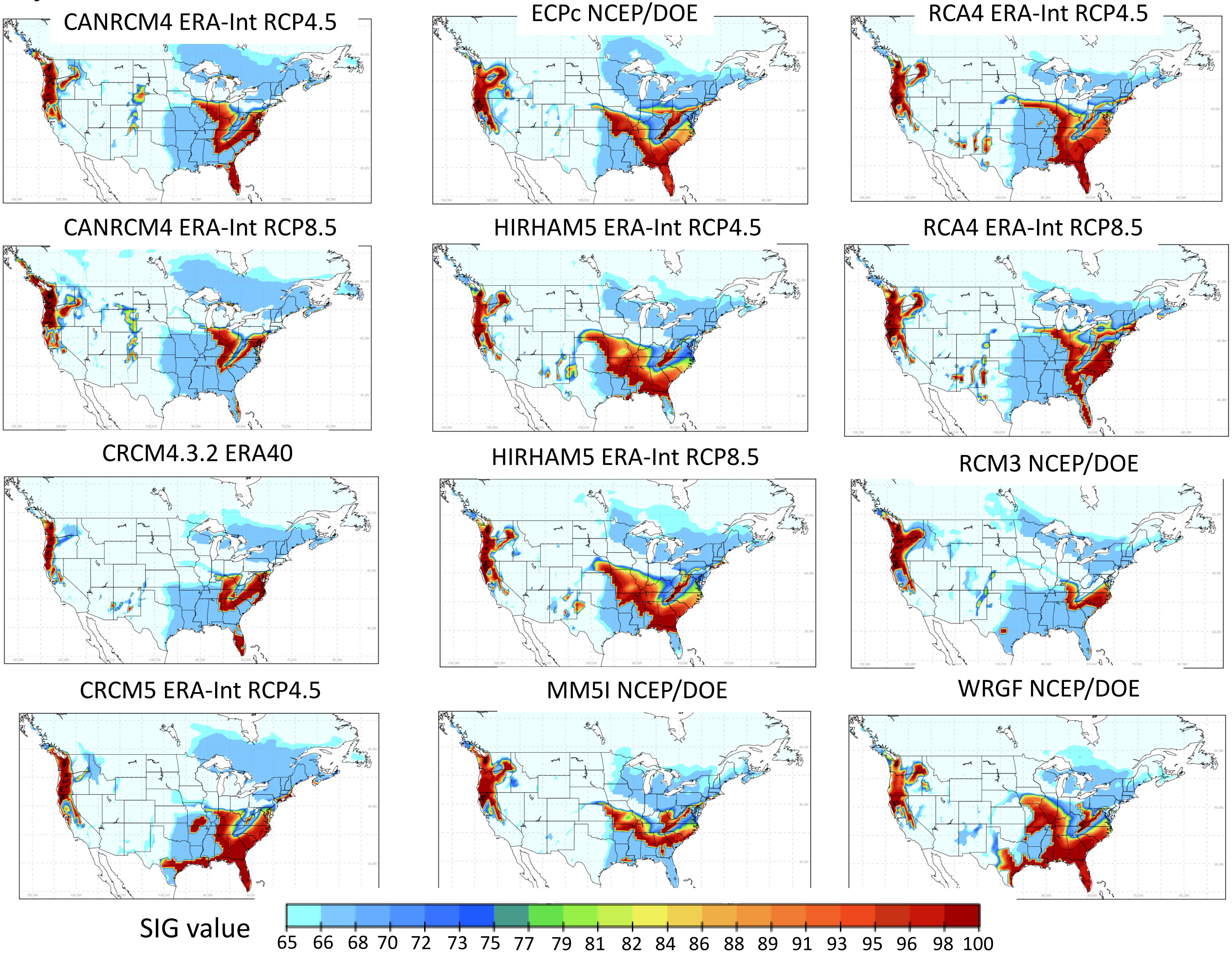

Supplement: Additional file 1: — The complete set of projections of climate suitability for Aedes albopictus in North America. Projected climate suitability according to each of the three climatic indicators OW, OWAT and SIG was obtained using output from nine Regional Climate Models (RCMs) as described in the main manuscript text. Maps of RCM-predicted current climate suitability obtained by forcing using global reanalysis, and future climate suitability for the time slices 2011-2040 and 2041-2070 (obtained using Representative Concentration Pathways RCP4.5 and RCP8.5) are shown. [file 13071_2014_532_MOESM1_ESM.pdf]
